# Isolation and biological evaluation of *N*-(4-aminocyclooctyl)-3, 5-dinitrobenzamide, a new semisynthetic derivative from the Mangrove-associated actinomycete *Pseudonocardia endophytica* VUK-10

**DOI:** 10.1007/s13205-016-0472-0

**Published:** 2016-07-27

**Authors:** Usha Kiranmayi Mangamuri, Muvva Vijayalakshmi, Sudhakar Poda, Bramanandam Manavathi, Bhujangarao Chitturi, Venkateswarlu Yenamandra

**Affiliations:** 1 Department of Botany and Microbiology, Acharya Nagarjuna University, Nagarjunanagar, Guntur, Andhra Pradesh 522510 India; 2 Department of Biotechnology, Acharya Nagarjuna University, Guntur, Andhra Pradesh 522510 India; 3 Molecular and Cellular Oncology Laboratory, Department of Biochemistry, School of Life Sciences, University of Hyderabad, Hyderabad, Telangana 500046 India; 4 Organic Chemistry Division-I, Indian Institute of Chemical Technology, Hyderabad, Telangana 500007 India

**Keywords:** *Pseudonocardia endophytica* VUK-10, Spectroscopy, *N*-(4-aminocyclooctyl)-3, 5-dinitrobenzamide, (3-((1H-indol-6-yl) methyl) hexahydropyrrolo [1, 2-a] pyrazine-1, 4-dione, Cytotoxicity, Antimicrobial activity

## Abstract

The present study was aimed to isolate novel bioactive compounds from actinomycetes species isolated from mangrove habitats. With this connection, *Pseudonocardia endophytica* (VUK-10) was isolated using dilution plate technique and was examined for its secondary metabolite profiling. After successive purification and spectroscopic characterization viz., FTIR, mass, NMR, DEPT, HMQC, HMBC, and COSY spectroscopy, two compounds were identified including a semi synthetic derivative *N*-(4-aminocyclooctyl)-3, 5-dinitrobenzamide (**1**), obtained from the precursor of novel natural product cyclooctane-1,4-diamine (**3**), along with a known compound 3-((1H-indol-6-yl) methyl) hexahydropyrrolo [1, 2-a] pyrazine-1, 4-dione (**2**). Anti cancer activities of the characterized compounds against in vitro cancerous cell line models, MDA-MB-231, OAW-42, HeLa, and MCF-7 reveal that HELA cells are most susceptible (IC_50_-10 nM compound **1** and **2**) followed by other studied cells. On the other hand, antibacterial and antifungal activities of the studied compounds against tested pathogens revealed that there is a significant antimicrobial activity with all the tested bacterial and fungal species. Moreover, compound **1** showed the lowest MIC values against *Streptococcus mutans* as 4  and 16 µg/ml for *Candida albicans*. In conclusion, the identified novel chemical compounds in the present study may have a potential application in anticancer therapy as well as to mitigate the bacterial and fungal pathogens thus to control the infectious diseases.

**Electronic supplementary material:**

The online version of this article (doi:10.1007/s13205-016-0472-0) contains supplementary material, which is available to authorized users.

## Introduction

The continuous search for the antimicrobial agents with affording protection, effective against clinical infections caused by Gram-negative bacteria, fungi, virus, and mycobacteira is at great demand. For many years, the focus on bioprospecting of novel secondary metabolites was from the terrestial environment but the marine habitat has been unlocked (Solanki et al. 2008). This is be cause of the imagination of scientists, that very few micro-organisms were present in the sea water due to extreme salinity which deemed to be a habitat unfavourable for many microbes dwelling. The belief of the scientist has been cornered due to the hypothesis of the researchers stating both the marine and terrestrial ecosystem conditions are extremely varied. The micro-organism dwelling in the marine environment should have possessed characteristics different from those of the terrestrial ecosystem that have a tendency to produce rare varied bioactive compounds (Davies et al. 2015). Extreme habitats have an immense potential as a source for novel microbes with taxonomic significance. These microbes serve as prolific producers of several important biomolecules due to their evolution and their metabolic adaptation in terms of the biochemical products such as enzymes and antibiotics (Satyanarayana et al. 2005). Marine microbes are virtually unlimited source of novel therapeutic compounds with medical application. Actinomycetes belong to the domain prokaryotes which inhabit diverse ecological niches and undoubtedly signifies their existence as psychrophilic, thermophilic, alkaliphilic, acidophilic, and halophilic (Goodfellow and Fiedler 2010). Actinom ycetes of the extremophilic category are a promising source for anti-microbial and cytotoxic compounds due to their unusual and diverse community and unexplored metabolic pathways existing in various genera at species variants (Shuvankar et al. 2015).

Microbial products being the natural source play a key role in the discovery and understanding of cellular pathways which are very vital in the drug discovery process. Many species belonging to the genera *Streptomyces* constitute 50 % of the soil microbial population and 75 % of molecules with antibiotic activity are produced by this genus (Piret and Demain 1988). Actinomycetes have been studied as potential producers of secondary metabolites with assorted chemical structures and biological properties. This led to search for useful products from rare genera of actinomycetes from unusual environments (Ellaiah et al. 2004; Khanna et al. 2011). The motive behind such strategies is to intensify the discovery of potential chemical moieties. Exploring new sources and introduction of effective antibiotics to fight the challenging and dreaded diseases is essential. Thus, searching for new actinomycetes from unexplored habitats constitute an essential component in natural product-based drug discovery (Clardy et al. 2006).

Antibiotics are one of our most important tools in fighting microbial infections and are greatly beneficial and improve the health profile of human life (Nayan and Shukla 2001). The antibiotic resistance growth coupled with apparent fall in the potential antibiotic number over the last two decades has raised concern about the future treatment of harmful and life threatening microbial infections. In addition, antibiotic resistance hindered the treatment and eradication of certain infecting pathogens affecting the immune system and the rise of different types of cancer has been greatly influencing the need to investigate new bioactive metabolites (Wise 2008; Talbot et al. 2006; Demain and Sanchez 2009).

Few reports have highlighted that the extreme habitats such as hot springs, deserts, marine ecosystem, and deep oceanic floors need to be explored for novel bioactive compounds (Ballav et al. 2012). The organisms isolated from such habitats serve as rich bioresource for the discovery of novel genera with novel chemical entities of biotechnological importance. Some of the natural compounds such as siderophores, indole acetic acid (Sadhegi et al. 2012) antagonistic metabolites, and antimicrobial metabolites (Dhanasekaran et al. 2005; Lakshmipathy and Kannabiran 2009) against clinical pathogens are obtained from the saline soil actinomycetes of the Indian subcontinent.

Mangroves are a specific woody plant community of intertidal coasts in the tropical and semitropical zones. They are considered as highly prolific and specific ecosystems and habitat to trap unexplored microbial diversity (Balagurunathan et al. 2010). There are ample evidences that the mangroves contain high populations of new and useful actinomycetes which become encouraging sources for bioactive metabolites (Kui et al. 2009). Recently, there has been elated interest in identification and biological evaluation of bioactive natural products from the rare actinomycetes (Hayakawa 2008). An intense literature survey reveals the bioactive metabolite profile of genus *Pseudonocardia*, covering antibacterial, antifungal, enzyme inhibitors, neuro protective, anti-*Helicobacter pylori* and cytotoxic compounds (Omura et al. 1979; Dekker et al. 1998; Maskey et al. 2003; Oh et al. 2009; Li et al. 2011; Gavin et al. 2012; Lee et al. 2012). As a part of our ongoing project of isolation of bioactive secondary metabolites from actinomycetes, we identified the strain *Pseudonocardia endophytica* VUK-10 isolated from Nizampatnam mangrove ecosystem (Ushakiranmayi et al. 2012) has been producing antimicrobial and cytotoxic active compounds. The present paper describes the extraction, purification, structural elucidation, and biological evaluation of the active metabolites produced by the supra said strain.

## Materials and methods

### Sample collection and physico-chemical analysis

**Fig. 1:**
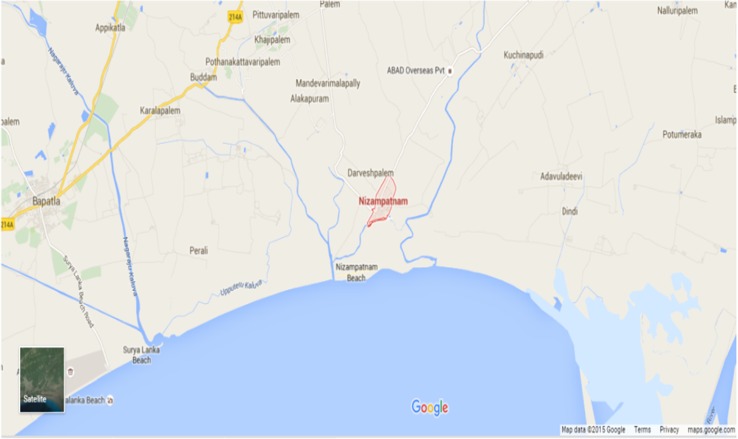
Sampling location of mangrove ecosystem of Nizampatnam

The sediment samples were collected at bimonthly intervals from April 2010 to March 2011 from different locations of mangrove ecosystem of Nizampatnam (Lat.15°54′0 N; Long. 80°40′0E) situated near the exit of Bay of Bengal along the south east coast of Andhra Pradesh, India (Fig. 1). Samples were collected from 6 to 10 cm depth and transported to the laboratory in sterile bags and later air dried at room temperature. The samples taken were analysed for physico-chemical properties such as moisture content (%), pH, temperature, organic carbon (%), and total nitrogen (%). To determine the moisture content, 10 g of sediment sample was dried in a hot air oven at 105 °C until a constant weight is obtained. The difference between the weights of pre-drying and post-drying was taken as the moisture content of the soil sample. The pH of sediment sample was determined according to Jackson (1973). Organic carbon was determined according to the method of Walkey and Black (1934). Nitrogen content was determined by Micro-Kjeld ahl method (AOAC 1995).

### Isolation

The air dried sediment sample was pre-treated with dry heat at 55 °C for 15 min and the treated sample was suspended in quarter strength ringer’s solution (1 g in 100 ml), homogenized by vortexing, and 0.1 ml of serially diluted sample was (10^−4^ dilution) spread over the surface of asparagine glucose agar medium containing 3 % NaCl supplemented with nalidixic acid (25 µg/ml) and secnidazole (25 µg/ml). After incubation for a week at 30 °C, distinct strain was selected and maintained by sub culturing on yeast extract-malt extract dextrose (YMD) agar medium at 4 °C for further study.

### Polyphasic taxonomy

The isolate was preliminarily characterized as described in the International *Streptomyces* Project (ISP) (Shirling and Gottlieb 1966). The cultural characteristics of the strain were studied on different media (Shirling and Gottlieb 1966). Micro morphology of the strain was examined by slide culture method (Williams and Cross 1971). Physiological characterization such as the effect of pH, temperature, and salinity tolerance was analysed (Ellaiah et al. 2004). Biochemical tests of the strain were also evaluated (Cowan 1974; Gordon 1966; Jones 1949; Waksman 1961). Carbohydrate utilization was determined by growing the strain on carbon utilization medium (ISP-9) (Pridham and Gottlieb 1948). Enzymatic profile of the strain was studied as described previously (Holding and Collee 1971; Gulati et al. 1997; Yeoh et al. 1985). Molecular genomic identification of the strain was carried out according to the procedure of Nilsson and Strom (2002).

### Extraction and secondary metabolite profiling

The seed broth was prepared by culturing *Pseudonocardia endophytica* VUK-10 on ISP-2 medium and incubated on a rotary shaker (250 rpm) at 35 °C for 48 h. Seed culture at the concentration of 10 % was transferred into fermentation broth consisting of glucose (8 %), soy-peptone (1 %), yeast extract (0.2 %), meat extract (0.1 %), CaCO_3_ (0.3 %), K_2_HPO_4_ (0.03 %), MgSO_4_ (0.1 %), FeSO_4_ (0.005 %), and NaCl (3 %) with pH adjusted to 7.0. The culture filtrates (40 L) obtained after cultivation of the strain for 96 h were extracted twice with ethyl acetate and concentrated in rotavap, freeze dried to yield a dark brown residue. The weight of the total crude extract was 5.5 g.

### Purification and structure elucidation

The ethyl acetate extract was subjected to Sephadex LH-20 gel filtration chromatography (36 × 2.5 cm, Sephadex G-15) using 1:1 DCM:MeOH (dichloromethane: methanol) as eluent yielded VII fractions. Based on the ^1^H-NMR spectral data and Bio-active screening, the fraction V (2.2 g) was selected for further study and subjected to silica gel column chromatography (25 × 5 cm, Silica gel 60, Merck) which afforded six fractions. Based on TLC monitoring and NMR spectral data, the sub fractions 2 and 5 were selected for further purification.

In the course of separation, initially the sub fraction 2 (369 mg) was subjected to dinitrobenzylation as it is difficult to isolate the compound, followed by purification on silica gel column chromatography using Dichloromethane: Acetone (92:8) as eluent and further purified by employing Chloroform: Isopropanol (80:20) yielded 54 mg of pure compound **1**. The sub fraction 5 (247 mg) was subjected to purification on silica gel column chromatography with dichloromethane: acetone (85:15) as eluent and further purified by employing chloroform: isopropanol (80:20) yielded 32 mg of pure compound **2**.

The structures of the isolated compounds were elucidated on the basis of 1D and 2D NMR spectral data correlations in combination with FT-IR spectra (Model: Thermo Nicolet Nexus 670 spectrophotometer with NaCl optics), Mass spectroscopy (EIMS; spectrum was measured on VG-7070H Micro mass spectrometer), 1D and 2D NMRs [^1^H, ^13^C, DEPT, HMQC, HMBC, and COSYNMR] were recorded on Avance 300 MHz spectrometer and CDCl_3_ was used as solvent.

### Test micro-organisms

Gram-positive bacteria: *Bacillus cereus* (MTCC 430), *Streptococcus mutans* (MTCC 497)*, Staphylococcus aureus* (MTCC 3160), *Staphylococcus epidermis* (MTCC 120), *Bacillus subtilis* (ATCC 6633), *Bacillus megaterium* (NCIM 2187); Gram-negative bacteria: *Escherichia coli* (ATCC 35218), *Pseudomonas aeruginosa* (ATCC 9027), *Proteus vulgaris* (MTCC 7299), *Serratia marcescens* (MTCC 118) *Xanthomonas campestris* (MTCC 2286), *Xanthomonas malvacearum* (NCIM 2954) and *Salmonella typhi* (ATCC 14028); Medically important dermatophytes: *Candida albicans* (ATCC 10231) and *Epidermophyton floccosum* (MTCC 145); Medically and agriculturally important filamentous fungi: *Aspergillus niger* (ATCC 1015), *Aspergillus flavus* (ATCC 9643), *Fusarium oxysporum* (MTCC 3075), *Fusarium solani* (MTCC 4634), *Penicillum citrinum* (MTCC 6489), *Verticillium alboatrum* and *Alternaria alternata* (MTCC 6572). The test micro organisms used in the present study were procured from ATCC, University Boulevard, Manassas, USA and MTCC, Chandigarh, NCIM, Pune, India and preserved at 4 °C.

### MIC assay

The antimicrobial spectra of the bioactive compounds produced by the strain were determined in terms of minimum inhibitory concentration (MIC) against a wide variety of bacteria and fungi using the broth dilution method (Andrews 2001). The purified compounds were dissolved in ethyl acetate while the antibiotics were dissolved in sterile distilled water to give stock concentrations of 2000 μg/ml. Twofold serial dilutions of the compounds and antibiotics were made with broth media at concentrations ranging from 1 to 1000 μg/ml. The test concentration of the compound along with the 100 μl of the microbial suspension (for bacteria 1 × 10^8^ and 1 × 10^7^ CFU/ml for yeast and fungi) was transferred aseptically to 10 ml of broth and incubated for 24 h at 37 °C for bacteria and 48–72 h at 28 °C for fungi. The growth was observed both visually and by measuring the OD at 600 nm against uninoculated broth. Triplicate sets of the tubes were maintained for each concentration of the test sample. The lowest concentration of the bioactive metabolites exhibiting significant antimicrobial activity against the test microorganisms was taken as the MIC of the compound.

### Cell lines and culture conditions

Cell lines for testing in vitro cytotoxicity: human breast adeno carcinoma cell line (MDA-MB-231), human cervical cancer cell line (HeLa), human ovarian cyst adenocarcinoma cell line (OAW-42) and human breast adenocarcinoma cell lines (MCF-7) (cell lines reported to be resistant to cancer drugs). The cell lines used in the present study were obtained from National Centre for Cell Science, Pune, India. Cell lines MDA-MB-231, HeLa, and OAW-42 were cultured on Dulbecco’s modified Eagle’s medium supplemented with fetal bovine serum (10 % (v/v)), l-glutamine (2 mM), penicillin (10 units/ml), and streptomycin (10 μg/ml), while breast cancer cell line MCF-7 was cultured on Roswell Park Memorial Institute medium 1640 supplemented with fetal bovine serum (10 %; (v/v)), l-glutamine (2 mM), penicillin (10 units/ml), and streptomycin (10 μg/ml), all in a humidified atmosphere (95 %) with 5 % of CO_2_ at 37 °C.

### MTT assay

The cytotoxicity of the compounds was assessed on the basis of the measurement of the in vitro growth in 96-well plates by cell-mediated reduction of tetrazolium salt to water insoluble formazan crystals, as per the micro culture MTT assay (Mosmann 1983). Cells were seeded in 96-well micro titer plates at a density of 5 × 10^3^ per well (100 µl) containing 0.1 ml of medium. After overnight incubation, the cells were treated with different test concentrations of bioactive compounds (10, 100, 1000, and 5000 nM) at identical conditions with three replicates of each concentration. After 24 h of incubation, the cell viability was assessed by adding 20 µl of MTT (5 mg/ml in PBS) per well and the plates were incubated at 37 °C for 4 h. The formazan crystals formed in the cells were dissolved with 100 µl of 0.1 % acidified isopropanol, and the rate of color development was measured at 570 nm using a micro plate reader. The IC_50_ values (50 % inhibitory concentration) of the compounds were calculated using Sigma Plot software with reference to that of Taxol as standard. All the experiments were carried out in triplicates.

## Results

### Physico-chemical analysis

Physico-chemical characteristics of sediment samples collected from Nizampatnam mangrove ecosystem were analysed and the parameters were recorded. The soil was slightly alkaline in nature with pH 7.6. The temperature of the site at the time of sample collection was measured at 32 °C. The moisture content of the soil sample was 15 %, organic carbon 7.2 mg/g, and the total nitrogen content was 4.5 µg/g. Soils with slightly alkaline or neutral pH together with 10–15 % moisture content and high organic content were reported to support high incidence of actinobacteria (De and Gupta 1993).

### Isolation and polyphasic taxonomy

**Fig. 2:**
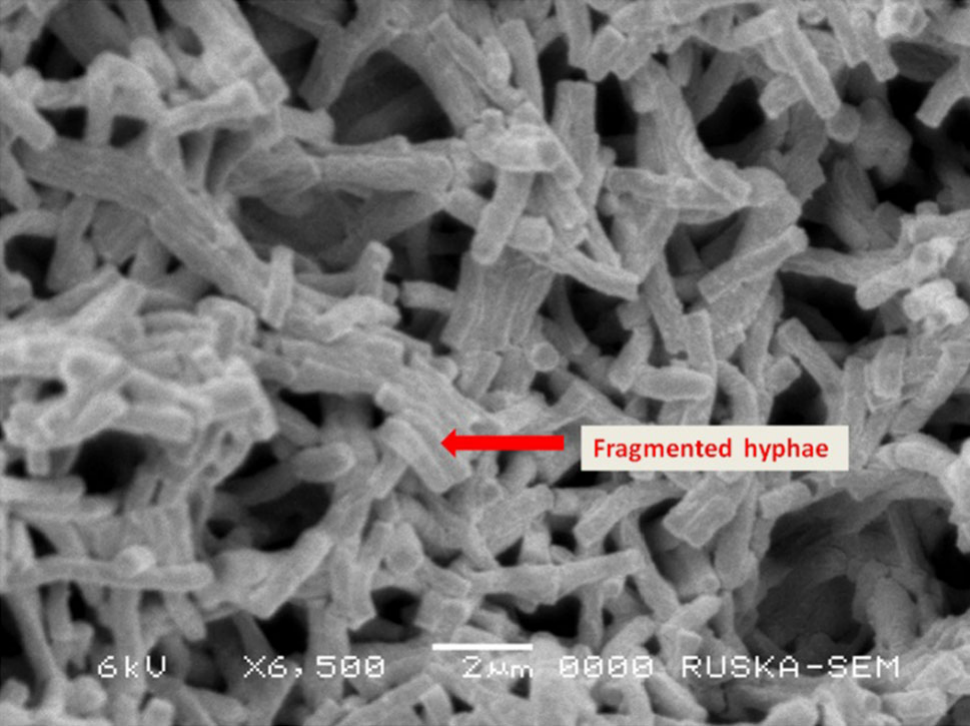
Scanning electron microscope image of *Pseudonocardia endophytica* VUK-10 (×6500)

Screening of soil samples from Nizampatnam mangrove ecosystem for potent actinomycetes led to the isolation of morphologically distinct actinomycete isolate VUK-10. The strain exhibited good growth on ISP-1, ISP-2, ISP-3, ISP-5, Czapek-Dox agar, and maltose tryptone agar, moderate on ISP-4 and ISP-7 agar, while growth was poor on nutrient agar media. Morphological and micro morphological observations of the strain revealed abundant aerial and vegetative hyphae which are well developed and fragmented to form rod shaped spores with smooth surface. Pigment production by the strain was not found on the culture media tested except melanin pigmentation on tyrosine (ISP-7) agar media. Detailed morphology of the strain was examined through scanning electron microscopy (SEM). Figure 2 depicts the SEM image of the strain at 6500× magnification. Growth of the strain occurred in the pH range of 6–9 with an optimum growth at pH 7. The temperature range for growth was 20–45 °C with the optimum temperature being 35 °C. The strain exhibited salt tolerance up to 10 % with optimum growth at 3 % NaCl. VUK-10 exhibited positive response to catalase production, starch hydrolysis and citrate utilization but negative for urease, hydrogen sulphide production, nitrate reduction, gelatine liquefaction, indole, methyl red, and Vogues-Proskauer tests. The strain efficiently utilized d-glucose, lactose, maltose, sucrose, galactose, fructose, and starch as carbon sources but not xylose, arabinose and mannitol. The strain could produce commercially important enzymes like amylase, cellulase, chitinase, and L-asparaginase but it was negative for protease, DNase, and RNase. 16S rDNA gene sequence of the isolate VUK-10 showed a close relation with *Pseudonocardia endophytica*. The rDNA sequence was deposited in the NCBI Gen Bank with an accession number JN087501 (Ushakiranmayi et al. 2012).

### Purification and structure elucidation

Culture filtrates obtained after 96 h fermentation were extracted with ethyl acetate and concentrated to yield a dark brown residue, which in turn was subjected to gel filtration chromatography using the solvent system of dichloromethane/methanol. Among the seven fractions collected, the fraction V exhibiting good antimicrobial activity was rechromatographed on a silica gel column and yielded six fractions. Based on TLC monitoring and NMR spectral data, the sub fractions 2 and 5 were selected for further purification. The sub fraction 2 (369 mg) was subjected to dinitrobenzylation followed by purification on silica gel column chromatography which yielded compound **1**. The sub fraction 5 was subjected to purification on silica gel column using different eluents which yielded pure compound **2**.

Compound (**1**) was obtained as white solid with m.p. 155–161 °C; $\left[ \alpha \right]_{\text{D}}^{ 2 5}$ +20 (c 0.5, CDCl_3_). Its molecular formula C_15_H_20_N_4_O_5_ was derived from HRESIMS *m/z* 359.1319 [M + Na]^+^ (calcd for C_15_H_20_NaN_4_O_5_ is 359.1331) and ^13^C NMR spectral data measurements. The structural elucidation of **1** was established by detailed interpretation of 1D and 2D NMR spectral data correlations (Table 1), including ^1^H NMR (Supplementary Fig. A), ^13^C NMR (Supplementary Fig. B), DEPT (Supplementary Fig. C), HMBC (Supplementary Fig. D), HSQC (Supplementary Fig. E) COSY (Supplementary Fig. F), and HRESIMS (Supplementary Fig. G). The ^13^C NMR and DEPT spectral data reveal the presence of 15 carbon resonances, including four of quarternary and six of methylene carbons along with two methane carbons acquired with nitrogen atoms. As the sub-fraction 5 (crude) was treated with 3,5-Dinitro benzoic acid in the presence of DCC/DMAP (solvent DCM) in DCM as solvent and stirred at 0 °C for 12 h followed by work-up procedures. The reaction mixture was monitored by TLC and purified on silica gel column chromatography to obtain product **1**, the ^13^C NMR spectrum of the resultant product (**1**) also had signals for tri-substituted aromatic ring carbons along with an amide carbonyl carbon between δ_C_ 120 ppm and δ_C_ 175 ppm. As the crude fraction (V) was treated with 3,5-dinitrobenzoic acid in DCC as solvent, the isolated semisynthetic product of the natural compound comprised with a 3,5-dinitrobenzoic acid ester residue in the structure. The molecular formula (C_15_H_20_N_4_O_5_) and spectral data account for six degrees of unsaturation including an aromatic ring and a carbonyl group; hence **1** is with di-substituted aliphatic monocyclic ring compound.

**Table 1:** 1D and 2D NMR data correlations of *N*-(4-aminocyclooctyl)-3,5-dinitrobenzamide

S. no	13C and (DEPT)	HSQC	^1^H-^1^H COSY	HMBC
1	56.0 (CH)	4.23 (tt, *J* = 3.59, 2.09 Hz)	2-H,	C-9, C-10, C-8, C-2
2	30.9 (CH_2_)	1.96 (d, *J* = 11.44 Hz) 1.74 (dt, *J* = 3.27, 12.45 Hz)	1-H, 3-H	C-8, C-1, C-3
3	32.1 (CH_2_)	1.48 (d *J* = 9.80 Hz) 0.84 (qd, *J* = 2.61, 12.09 Hz)	4-H, 2-H	C-4, C-5, C-2
4	50.6 (CH)	3.51 (m)	3-H	C-3, C-5
5	25.5 (CH_2_)	1.71 (m) 1.19 (q, *J* = 13.07 Hz)	–	C-7, C-2, C-4
6	25.4 (CH_2_)	1.56 (d, *J* = 13.07 Hz) 1.03 (m)	–	C-3, C-5
7	24.9 (CH_2_)	1.58 (m) 1.19 (q, *J* = 13.07 Hz)	–	C-5, C-2, C-4
8	26.2 (CH_2_)	1.87 (d, *J* = 13.07 Hz) 1.39 (q, *J* = 13.07 Hz)	8-H↔ 8-H	C-2, C-1,C-7
9	165.0 (Cq)	–	–	–
10	153.9 (Cq)	–	–	–
11	127.8 (2CH)	8.72 (d, *J* = 2.31 Hz)	–	C-13, C-15, C-12, C-9
12	148.4 (Cq)	–	–	–
13	120.3 (CH)	9.09 (s)	–	C-11, C-12, C-14, C-15
14	148.4 (Cq)	–	–	–
15	127.8 (2CH)	8.72 (d, *J* = 2.31 Hz)	–	C-13, C-11, C-12 (14), C-9
16	(N-H)	5.85 (d, *J* = 7.84 Hz)	–	C-3, C-4, C-10

**Fig. 3:**
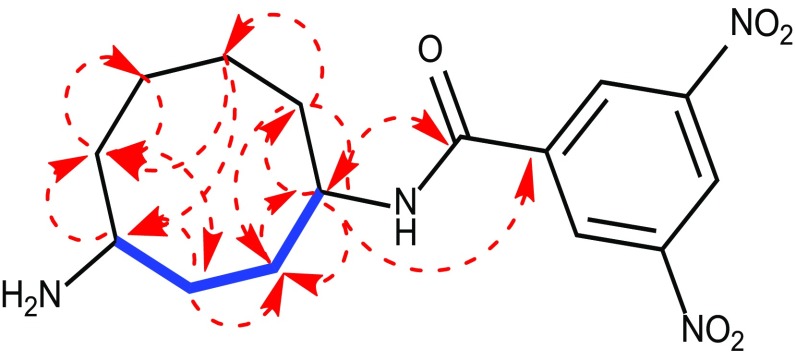
Important COSY (–) and HMBC (→) correlations of semisynthetic compound *N*-(4-aminocyclooctyl)-3,5 dinitrobenzamide

**Fig. 4:**
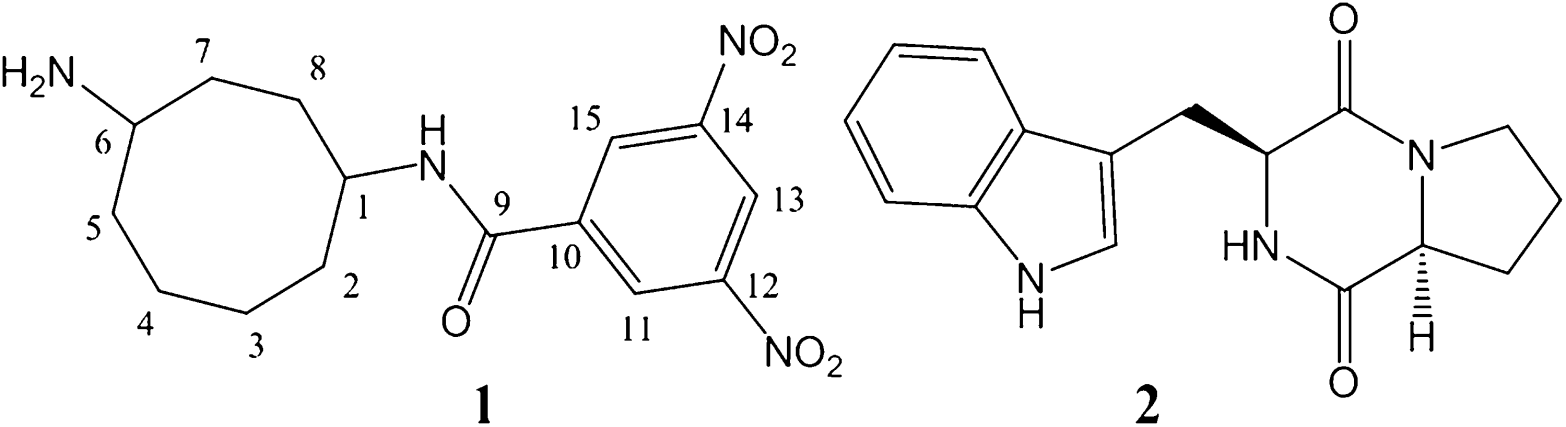
Structures of the compounds **1** and **2** isolated from *Pseudonocardia endophytica* VUK-10

The ^1^H-^1^H COSY spectrum of **1** was consistent showing the linear correlation between 1-H (*d*_H_ 4.23, tt), 2-2H (*d*_H_ 1.96, d and 1.74, dt), 3-2H (*d*_H_ 1.48, d and 0.84, qd) and 4-H (*d*_H_ 3.51, m) protons. The HMBC correlations of (**1**) consistently suggesting cyclic-octane ring formulation (1-H/C2, C8, C9, C10; 2-H/C8, C1; 3-H/C2, C4, C5; 4-H/C3, C5; 5-H/C-2, C-4, C-7; 6-H/C-3, C-5; 7-H/C-2, C-4, C-5 and 8-H/C-1, C-2, C-7) in addition to the dinitrobenzyl group in the structure (Fig. 3). Meticulous analysis of 1D and 2D NMR spectral data correlations confirmed that, (**1)** is a novel cyclooctane-1, 4-diamine carbon skeleton derivative (Fig. 4).

Compound (**2**) was obtained as white amorphous solid which was completely soluble in DMSO, methanol, ethanol, *N*–*N*-dimethyl formamide, and ethyl acetate but insoluble in water and *n*–hexane. The ^1^H-NMR (300 MHz, CDCl_3_) spectrum of the compound **2** showed signals at δ 8.46 (s, 1H); 7.57 (d, 1H, *J* = 7.7 Hz); 7.37 (d, 1H, *J* = 8.1 Hz); 7.22 (t, 1H, *J* = 7.1 Hz); 7.16-7.08 (m, 2H); 5.84 (s, 1H); 4.37 (dd, 1H, *J* = 2.6, 10.5 Hz); 4.06 (t, 1H, *J* = 6.6 Hz); 3.72 (dd, 1H, *J* = 3.3, 14.9 Hz); 3.66–3.48 (m, 2H); 2.96 (dd, 1H, *J* = 10.5, 14.9 Hz); 2.35–2.27 (s, 1H); 2.06–1.82 (m, 3H) (Supplementary Fig. H), while ^13^C (75 MHz, CDCl_3_) exhibited signals at (75 MHz, CDCl_3_) δ 169.4, 165.4, 136.5, 126.6, 123.4, 122.5, 119.7, 118.4, 111.5, 109.6, 59.1, 54.5, 45.3, 28.1, 26.7, and 22.5 (Supplementary Fig. I). Mass spectrometric (EIMS) analysis of the compound gave a molecular ion peak at *m*/*z* 306 (M + 23) (Supplementary Fig. J). In IR spectrum, the compound exhibited absorption bands at *V*_max_ 3280, 2924, 1658, 1430, 1103, and 745 cm^−1^ (Supplementary Fig. K). On the basis of spectral data (C_16_H_17_N_3_O_2_) analysis and literature survey (Ivanova et al. 2003), the compound **2** was identified as (3-(1H-indol-6-yl) methyl) hexahydropyrrolo [1, 2-a] pyrazine-1, 4-dione (Fig. 4).

### MIC assay

Antibacterial activities of the bioactive compounds (**1** and **2**) in terms of MIC are shown in Table 2. Bioactive compounds exhibited antibacterial activity against a variety of Gram-positive and Gram-negative bacteria for which the MIC values ranged from 4 to 64 μg/ml. Among the facultative and pathogenic Gram-positive bacteria, the compound **1** was active against all the bacteria tested and the best activity of this compound was recorded against *Streptococcus mutans* (4 μg/ml) followed by *Bacillus cereus* (8 μg/ml). Compound **2** presented highest activity against *Staphylococcus aureus* (16 μg/ml) followed by *Bacillus megaterium* (32 μg/ml). Of the Gram-negative bacteria, the micro-organisms that presented highest sensitivity towards compound **1** was *Pseudomonas aeruginosa* (16 μg/ml) followed by *Salmonella typhi*, *Escherichia coli,* and *Xanthomonas malvacearum* (32 μg/ml). Compound **2** recorded highest activity against *Escherichia coli* and *Xanthomonas malvacearum* (16 μg/ml). Tetracycline served as positive control for bacteria. Compared to standard drug tetracycline, compound **1** displayed high sensitivity against *Streptococcus mutans* and *Staphylococcus aureus,* while compound **2** recorded similar sensitivity like positive control against *Serratia marcescens* (Table 2). Tetracycline, in other cases showed good antibacterial activity over the metabolites of the strain.

**Table 2:** Minimum inhibitory concentration (MIC) of bioactive compounds isolated from *Pseudonocardia endophytica* VUK-10 [MIC-(μg/ml)] against test bacteria

Test microorganisms	Compound **1**	Compound **2**	Tetracycline
*S. aureus*	16 ± 0.02	16 ± 0.01	32 ± 0.03
*S. mutans*	4 ± 0.03	64 ± 0.00	32 ± 0.03
*S. epidermis*	16 ± 0.01	64 ± 0.02	16 ± 0.03
*X. campestris*	64 ± 0.00	32 ± 0.03	16 ± 0.03
*X. malvacearum*	32 ± 0.02	16 ± 0.01	8 ± 0.01
*B. subtilis*	16 ± 0.03	64 ± 0.03	32 ± 0.02
*B. megaterium*	16 ± 0.02	32 ± 0.02	16 ± 0.03
*B. cereus*	8 ± 0.01	64 ± 0.01	8 ± 0.02
*E. coli*	32 ± 0.01	16 ± 0.01	8 ± 0.01
*P. aeruginosa*	16 ± 0.02	64 ± 0.02	8 ± 0.03
*S. marcescens*	64 ± 0.01	32 ± 0.03	32 ± 0.01
*P. vulgaris*	64 ± 0.00	64 ± 0.01	16 ± 0.03
*S. typhi*	32 ± 0.03	64 ± 0.02	8 ± 0.02

Values are mean ± standard deviation (*n* = 3). Compound **1**: *N*-(4-aminocyclooctyl)-3, 5-dinitrobenzamide. Compound **2**: (3-((1H-indol-6-yl) methyl) hexahydropyrrolo [1, 2-a] pyrazine-1, 4-dione. Antibiotic: tetracycline

Antifungal activity against dermatophytes and filamentous fungi and corresponding MIC values are recorded in Table 3. The bioactive compounds showed significant antifungal activity against the dermatophytes (*Candida albicans* and *Epidermophyton floccosum*). Among the filamentous fungi tested, *Aspergillus niger* recorded sensitivity of 32 μg/ml followed by *Aspergillus flavus*, *Penicillium citrinum,* and *Verticillium alboatrum* (64 μg/ml) towards compound **1**. Compound **2** was active against *Aspergillus flavus* and *Fusarium solani* at 64 μg/ml and for this compound *Verticillium alboatrum* recorded no activity up to 512 μg/ml. Both compounds recorded lower antifungal activity than the standard fungicide amphotericin-B against fungi.

**Table 3:** Minimum inhibitory concentration (MIC) of bioactive compounds isolated from *Pseudonocardia endophytica* VUK-10 [MIC-(μg/ml)] against dermatophytes and fungi

	Compound **1**	Compound **2**	Antifungal agent
Dermatophytes			
*Candida albicans*	16 ± 0.03	64 ± 0.02	16 ± 0.01
*Epidermophyton floccosum*	32 ± 0.02	32 ± 0.00	16 ± 0.01
Fungi			
*Aspergillus niger*	32 ± 0.02	128 ± 0.03	16 ± 0.02
*Aspergillus flavus*	64 ± 0.00	64 ± 0.02	8 ± 0.00
*Fusarium oxysporum*	128 ± 0.01	256 ± 0.01	16 ± 0.00
*Fusarium solani*	128 ± 0.02	64 ± 0.02	32 ± 0.01
*Penicillium citrinum*	64 ± 0.00	256 ± 0.03	8 ± 0.01
*Verticillium alboatrum*	64 ± 0.03	>512 ± 0.02	64 ± 0.02
*Alternaria alternata*	128 ± 0.03	128 ± 0.01	32 ± 0.01

Values are mean ± standard deviation (*n* = 3). Compound **1**: *N*-(4-aminocyclooctyl)-3, 5-dinitrobenzamide. Compound **2**: 3-((1H-indol-6-yl) methyl) hexahydropyrrolo [1, 2-a] pyrazine-1, 4-dione. Antifungal agent: griseofulvin against dermatophytes and amphotericin-B against fungi

### MTT assay

The in vitro cytotoxicity evaluated against a panel of human tumor cell lines indicated that both the compounds had similar level of anti tumor activity against MDA-MB-231, HeLa, OAW-42, and MCF-7 cell lines.

**Fig. 5:**
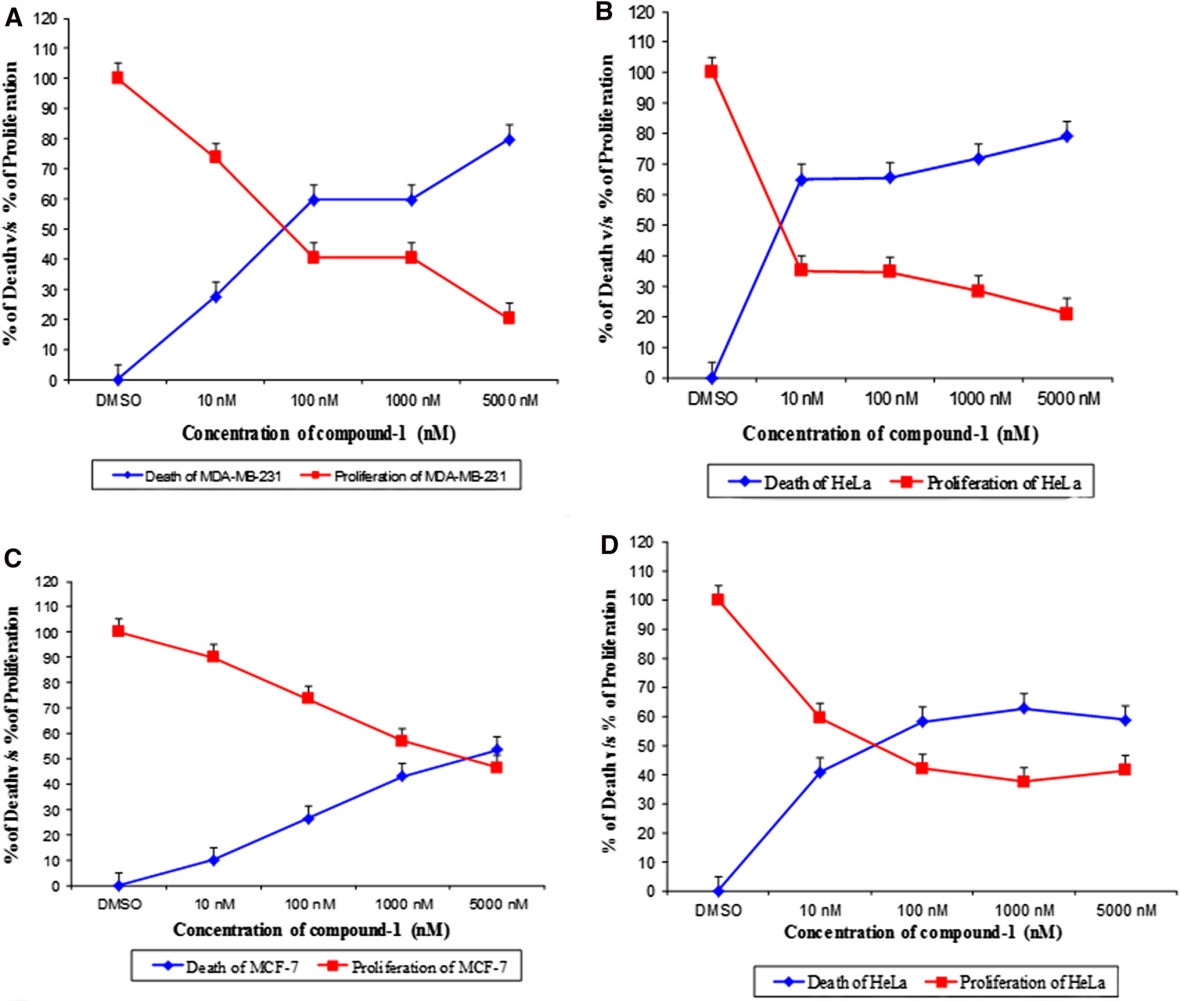
Dose response curve of Compound **1** on the growth of **a** MDA-MB-231, **b** HeLa, **c** MCF-7 and **d** OAW-42 cell lines

The activity of compound **1** against MDA-MB-231, HeLa, MCF-7, and OAW-42 cell lines is presented in Fig. 5a–d. Compound **1** exhibited potent cytotoxicity when evaluated against MDA-MB-231, HeLa, MCF-7, and OAW-42 cell lines displaying IC_50_ values of 100 nM (59.6, 58 %) (MDA-MB-231) (OAW-42), 10 nM (64.9 %) (HeLa), and 5000 nM (53.4 %) (MCF-7).

**Fig. 6:**
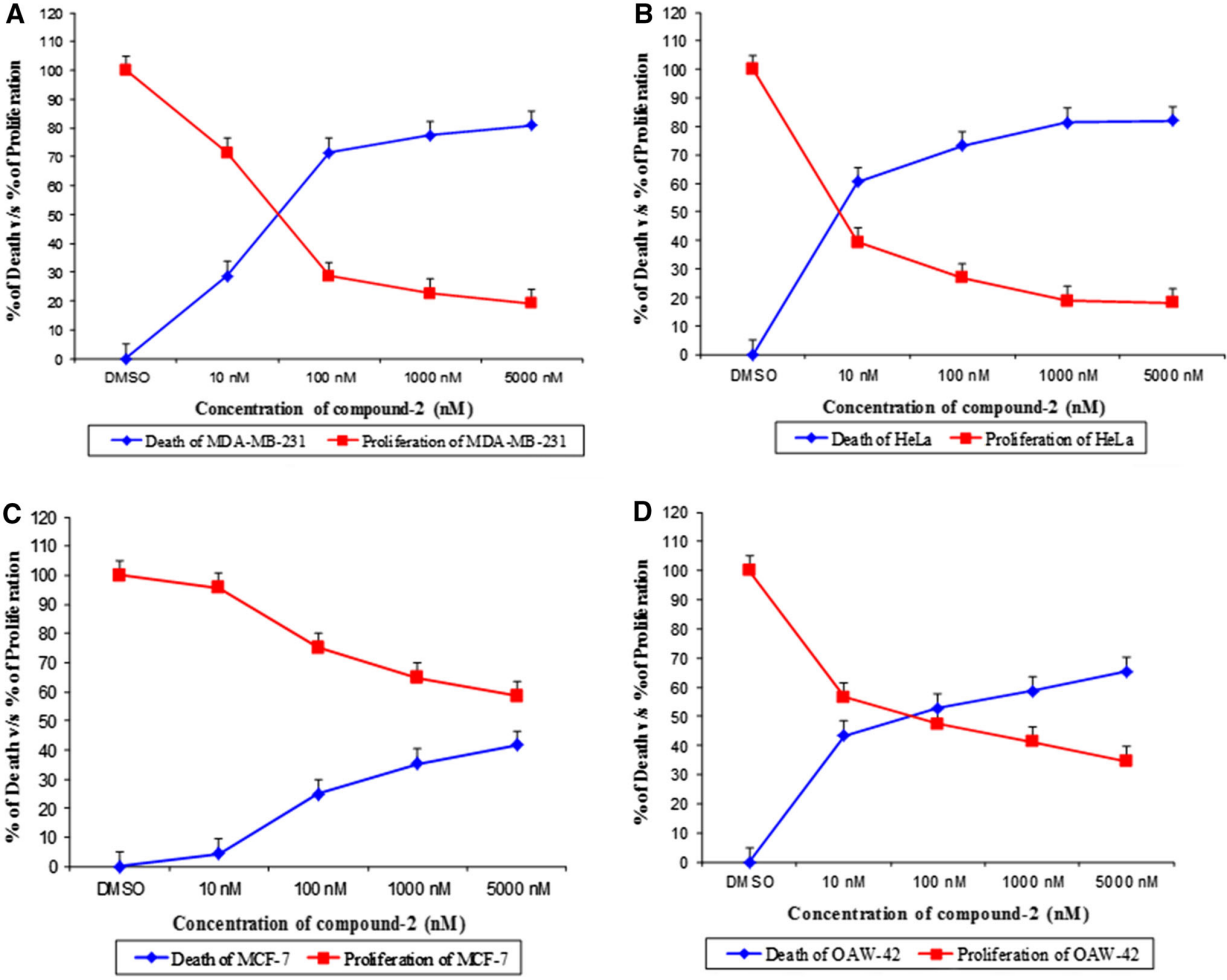
Dose response curve of Compound **2** on the growth of **a** MDB-MB-231, **b** HeLa, **c** MCF-7 and **d** OAW-42 cell lines

The activity of compound **2** against MDA-MB-231, HeLa, MCF-7, and OAW-42 cell lines is presented in Fig. 6a–d. Compound **2** showed significant cytotoxicity with MDA-MB-231, HeLa, MCF-7, and OAW-42 cell lines. The cell lines exhibited IC_50_ values of 100 nM (71.5 %, 52.7 %) (MDA-MB-231) (OAW-42), 10 nM (60.6 %) (HeLa), and 5000 nM (41.6 %) (MCF-7), with Taxol an anti-cancer drug used as standard that recorded an IC_50_ value of 10 nM (59, 60, 57, and 63 %) against MDA-MB-231, HeLa, OAW-42, and MCF-7 cell lines.

## Discussion

Recent research efforts have focused on the exploration of under explored habitats to discover novel bioactive secondary metabolites from the autochthonous microbiota (Omar et al. 2015). Natural products are the important assuring source for novel bioactive molecules especially antimicrobial compounds over combinatorial chemistry and fragment based drug designing that devote very little to corner the spread of the antibiotic resistant pathogens (Shuvankar et al. 2015). Antibiotics are the group of important bioactive compounds for the treatment of infectious diseases. Natural products remain as most prominent source for the exploration of uncommon innovative bioactive molecules especially antimicrobial compounds due to the fact that the spread of the antibiotic resistant pathogens cannot be counteracted and reversed since combinatorial chemistry and fragment-based drug designing contribute very little (Goodfellow and Fiedler 2010; Mikhail et al. 2015). Among the different niches, mangrove forests are exclusively rare niches which harbor a diverse range of microbes (Farshid and Faranak 2015). Actinomycetes are the predominant members of marine microbial communities and are also known to adapt to harsh environmental conditions (Augustine et al. 2012).

**Fig. 7:**
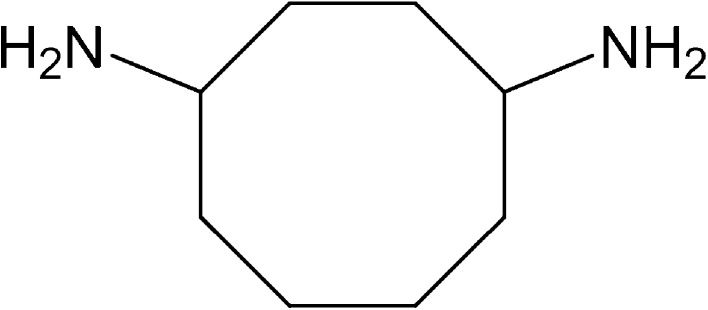
Compound **3** (not been isolated purely) is the precursor of semi synthetic compound **1**

In the present screening programme undertaken for exploration of the novel bioactive secondary metabolites from Nizampatnam mangrove ecosystem, a potent isolate with high antimicrobial and cytotoxic activities identified as *Pseudonocardia endophytica* VUK-10 was isolated. A semisynthetic derivative (*N*-(4-aminocyclooctyl)-3, 5-dinitrobenzamide) along with a known compound (3-(1H-indol-6-yl) methyl) hexahydropyrrolo [1, 2-a] pyrazine-1, 4-dione) was isolated from the fermentation broth of the strain grown on glucose-soy peptone broth. The precursor of the semisynthetic compound **1** could be cyclooctane-1, 4-diamine (**3**) (Fig. 7). A quick SciFinder search reveals that this is an unknown compound that has not been identified as a metabolite from any microorganisms.

The compound **2**, a non-ribosomal peptide is indistinguishable with cyclo (tryptophanyl prolyl), a cyclic dipeptide between the amino acids tryptophan and proline. Cyclic dipeptides (CDPs) are commonly biosynthesized from amino acids by different organisms, including mammals, and are considered to be secondary functional metabolites or side products of terminal peptide cleavage (Maristela and Ivone 2007). CDPs are more than simple curiosities and are well known for their economically beneficial biological activities (Prasad 1955). The therapeutic potential of these compounds reflects their antibiotic, antiviral, and antitumor properties and various cyclic dipeptides are detectable in protein and polypeptide hydrolysates as well as in cultures of yeasts, lichens, and molds (Otteinheijm et al. 1976; Graz et al. 1999; Katrin et al. 2002). The isolation of compound **2** was previously reported as a natural product from two *Penicillium* strains (Paterson et al. 1990).

Our results showed that compound **1** has a good potential inhibitor against *Streptococcus mutans* (responsible for human dental decay), *Pseudomonas aeruginosa* (causes pneumonia, bacteremia, and endocarditis), *Candida albicans* (causes oral thrush and vaginal infection) and *Aspergillus niger* (causes black mold on grapes, onions, and peanuts). Compound **2** showed to have antimicrobial activity against *Staphylococcus aureus* (causes impetigo, carbuncles, and abscesses), *Escherichia coli* (causes cholecystitis, bacteremia, cholangitis, and urinary tract infection), *Xanthomonas malvacearum* (causes angular leaf spot of cotton) and *Epidermophyton floccosum* (causes tinea pedis, tinea cruris, and tinea corporis).

The potential of compounds **1** and **2** was investigated to inhibit cancer cell growth in MDA-MB-231, HeLa, OAW-42, and MCF-7 cell lines. Our results showed that compounds **1** and **2** displayed similar level of significant cytotoxicity with variation in percentage of cell line inhibition against MDA-MB-231, HeLa, OAW-42, and MCF-7 cell lines. To the best of our knowledge, anti cancer activity of compounds **1** and **2** against the above said cell lines are reported here for the first time. The data reported in this paper clearly indicate that our compounds exhibit significant anti cancer activities at nanomolar concentrations. Contemporary identification of primary and secondary metabolites from taxonomically exclusive population of actinomycetes disclosed that these microbes could add a new element to microbial natural product research (Jicheng et al. 2015).

## Conclusion

Based on the distribution, actinomycetes have been validated as an important source of bioactive metabolites. The present study is concluded that actinomycetes strain *Pseudonocardia endophytica* VUK-10 with an interesting bioactivity profile has been exploited vividly to study their bioactive metabolites. The results of the present study are amazing and the compounds **1** and **2** exhibited strong effects against test microorganisms and cancer cell lines even in impressive low concentrations. Therefore, the bioactive compounds can be ideal ammunition to use as a potential antimicrobial and anticancer agents and trusting it as the first report of isolation and characterization of compounds **1** and **2** from the genus *Pseudonocardia.*

## Electronic supplementary material

Below is the link to the electronic supplementary material.

Supplementary material 1 (DOCX 2444 kb)
