# Supplementary material for: Isolation and biological evaluation of N-(4-aminocyclooctyl)-3, 5-dinitrobenzamide, a new semisynthetic derivative from the Mangrove-associated actinomycete Pseudonocardia endophytica VUK-10
Source: 3 Biotech. 2016 Jul 27;6(2):158. doi: 10.1007/s13205-016-0472-0 (PMC4963327; doi:10.1007/s13205-016-0472-0)
Supplement: Supplementary file 1 — Supplementary material 1 (DOCX 2444 kb) [file 13205_2016_472_MOESM1_ESM.docx]

**Supplementary Information**

**Isolation and Biological Evaluation of N-(4-aminocyclooctyl)-3, 5-dinitrobenzamide, a new semi synthetic derivative from the Mangrove associated actinomycete *Pseudonocardia endophytica* VUK-10**

**Usha Kiranmayi Mangamuri^1^, Muvva Vijayalakshmi^1^*, Sudhakar Poda^2^, Bramanandam Manavathi^3^, Bhujangarao Chitturi^4^ and Venkateswarlu Yenamandra^4^**

***^1^Department of Botany & Microbiology, Acharya Nagarjuna University, Guntur-522510, Andhra Pradesh, India. Email:*** [***drmangamuri@gmail.com***](mailto:drmangamuri@gmail.com)

***^2^Department of Biotechnology, Acharya Nagarjuna University, Guntur-522510, Andhra Pradesh, India. Email:*** [***sudhakarpodha@gmail.com***](mailto:sudhakarpodha@gmail.com)

***^3^Molecular and Cellular Oncology Laboratory, Department of Biochemistry, School of Life sciences, University of Hyderabad, Hyderabad-500046, India. Email:*** [***manavbrahma@yahoo.com***](mailto:manavbrahma@yahoo.com)

***^4^Organic Chemistry Division-I, Indian institute of Chemical Technology, Hyderabad-500007, India. Email:*** [***cbraoiict@gmail.com***](mailto:cbraoiict@gmail.com)

****Corresponding author:***

***Prof. M. Vijayalakshmi***

***Dean, Life Sciences***

***Acharya Nagarjuna University***

***Nagarjunanagar, Guntur-522510***

***Andhra Pradesh, India***

***Email:*** [***profmvl@gmail.com***](mailto:profmvl@gmail.com)

***Phone No: +91-9440870026***

***Fax No: +91-0863-2293378***

#


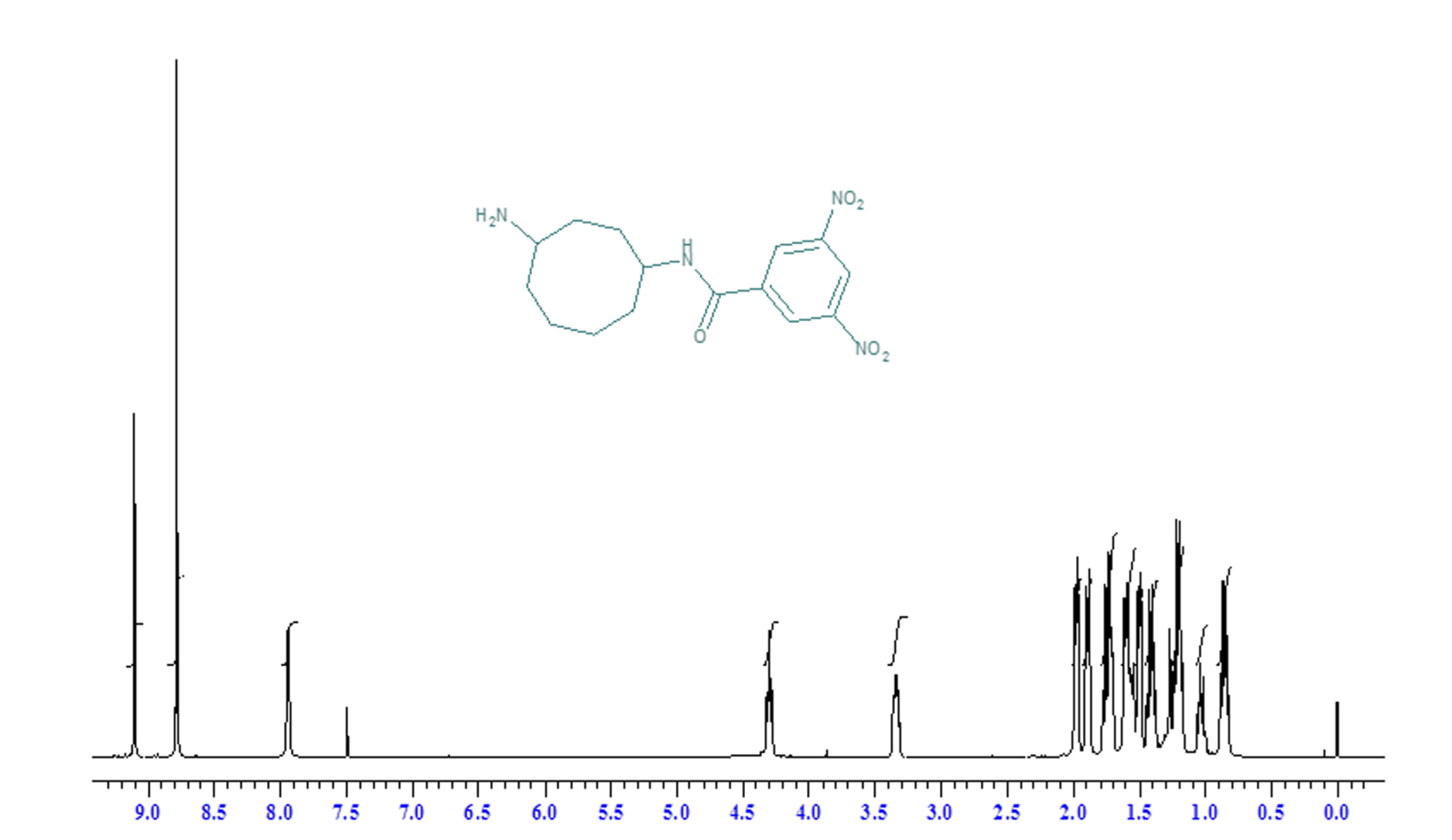


**Supplementary Fig. A**: ^1^H NMR spectrum (CDCl_3_, 600 MHz) of N-(4-aminocyclooctyl)-3, 5-dinitrobenzamide (1).


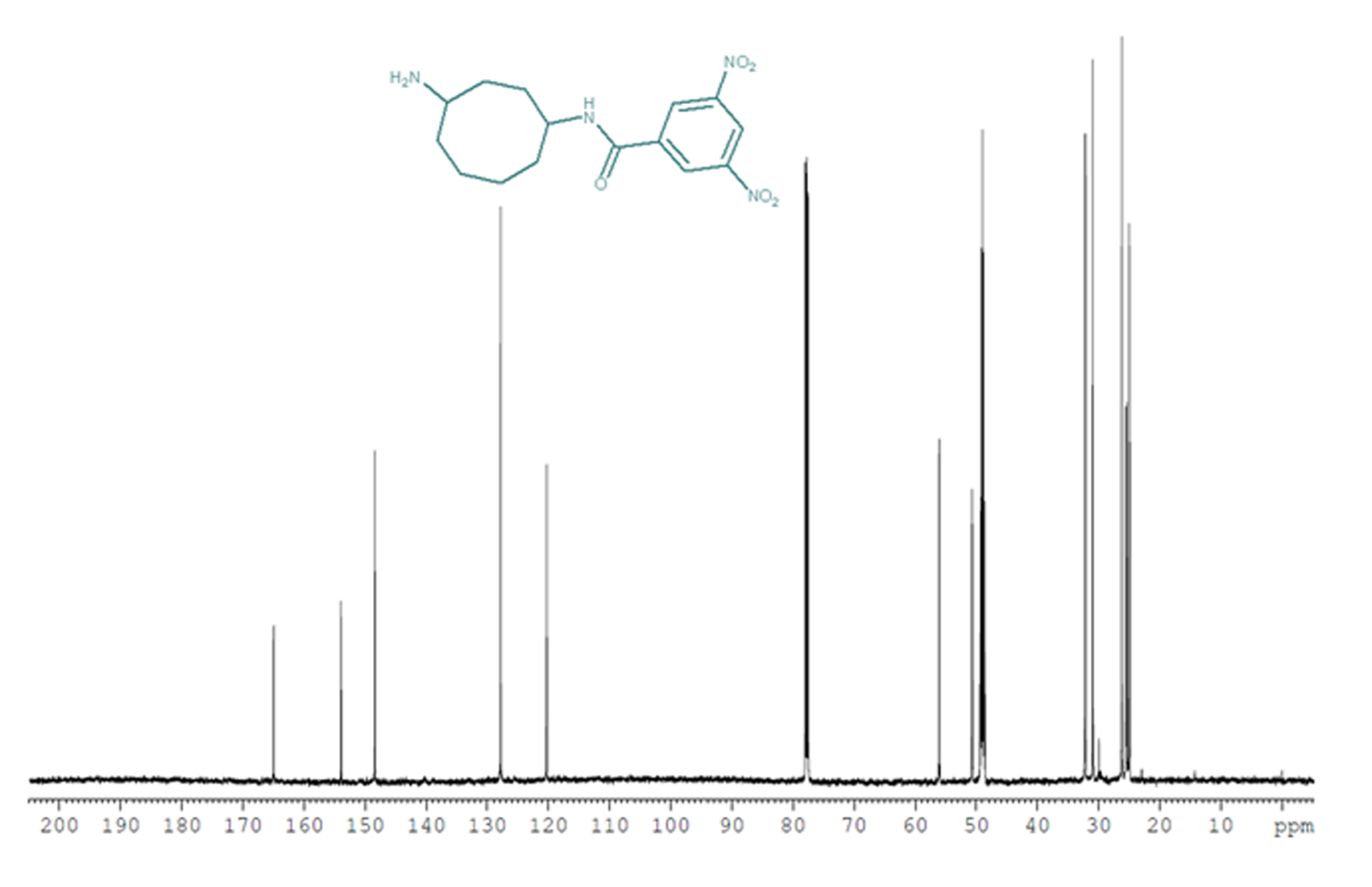


**Supplementary Fig. B**: ^13^CNMR spectrum (CDCl_3_, 600 MHz) of N-(4-aminocyclooctyl)-3, 5-dinitrobenzamide (1).


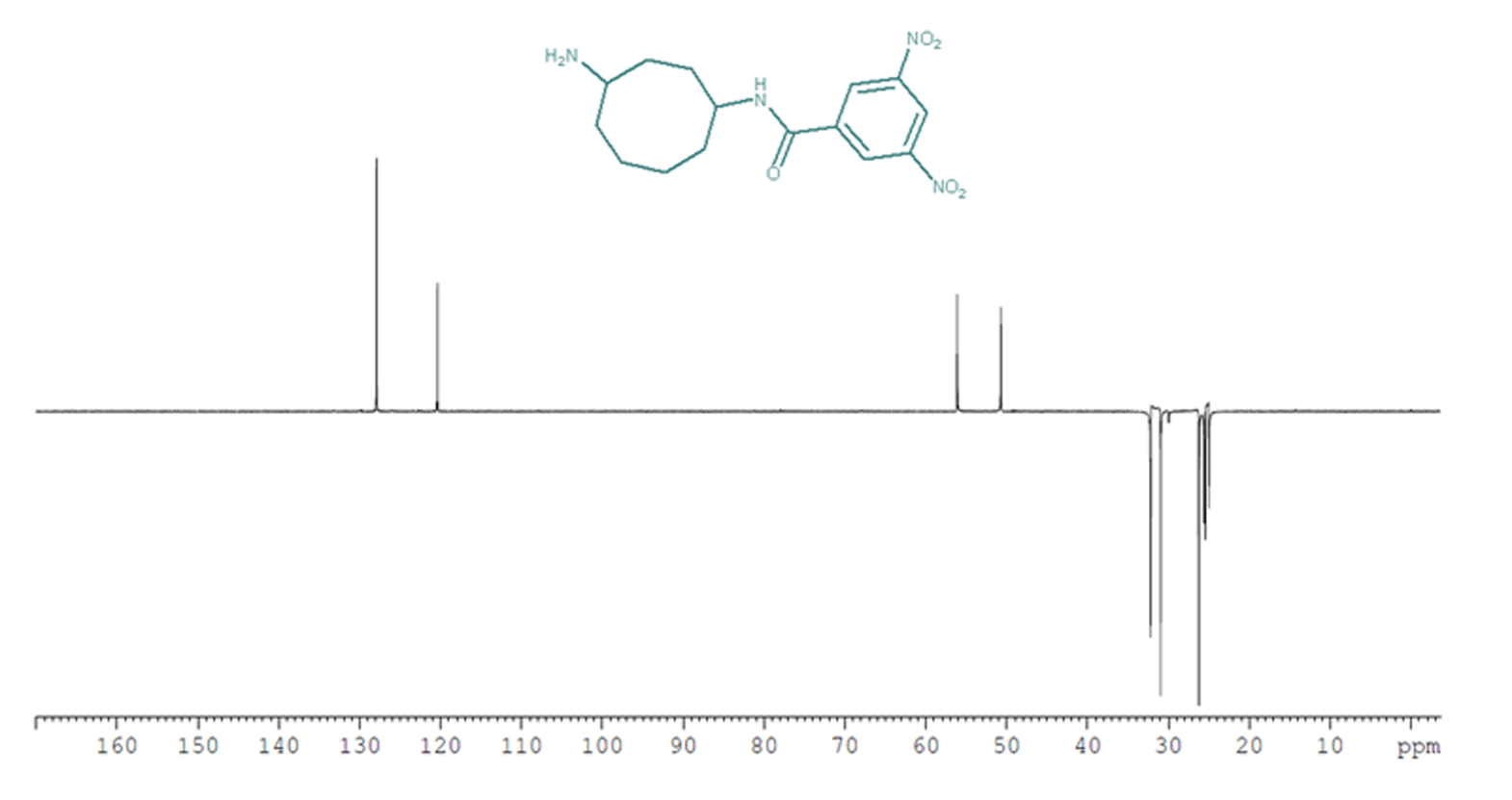


**Supplementary Fig. C**: DEPT spectrum (CDCl_3_, 600 MHz) of N-(4-aminocyclooctyl)-3, 5dinitrobenzamide (**1**).


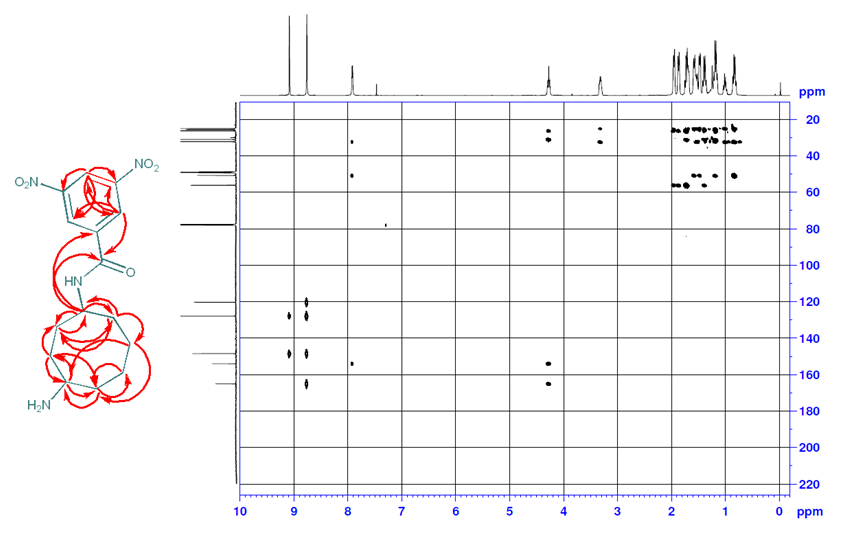


**Supplementary Fig. D :** HMBC spectrum (CDCl_3_, 600 MHz) of N-(4-aminocyclooctyl)-3, 5-dinitrobenzamide (**1**).


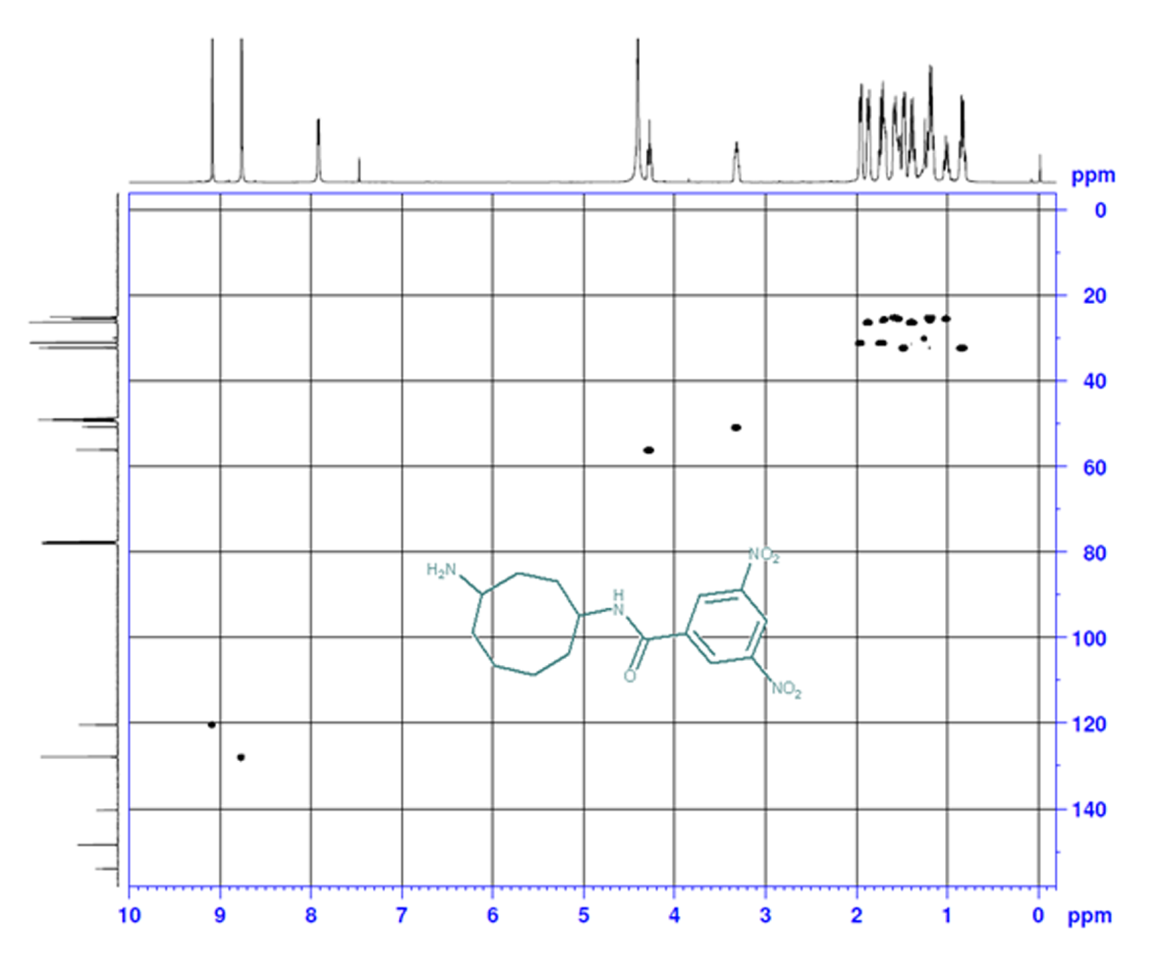


**Supplementary Fig. E**: HSQC spectrum (CDCl_3_, 600 MHz) of N-(4-aminocyclooctyl)-3, 5-dinitrobenzamide (1).


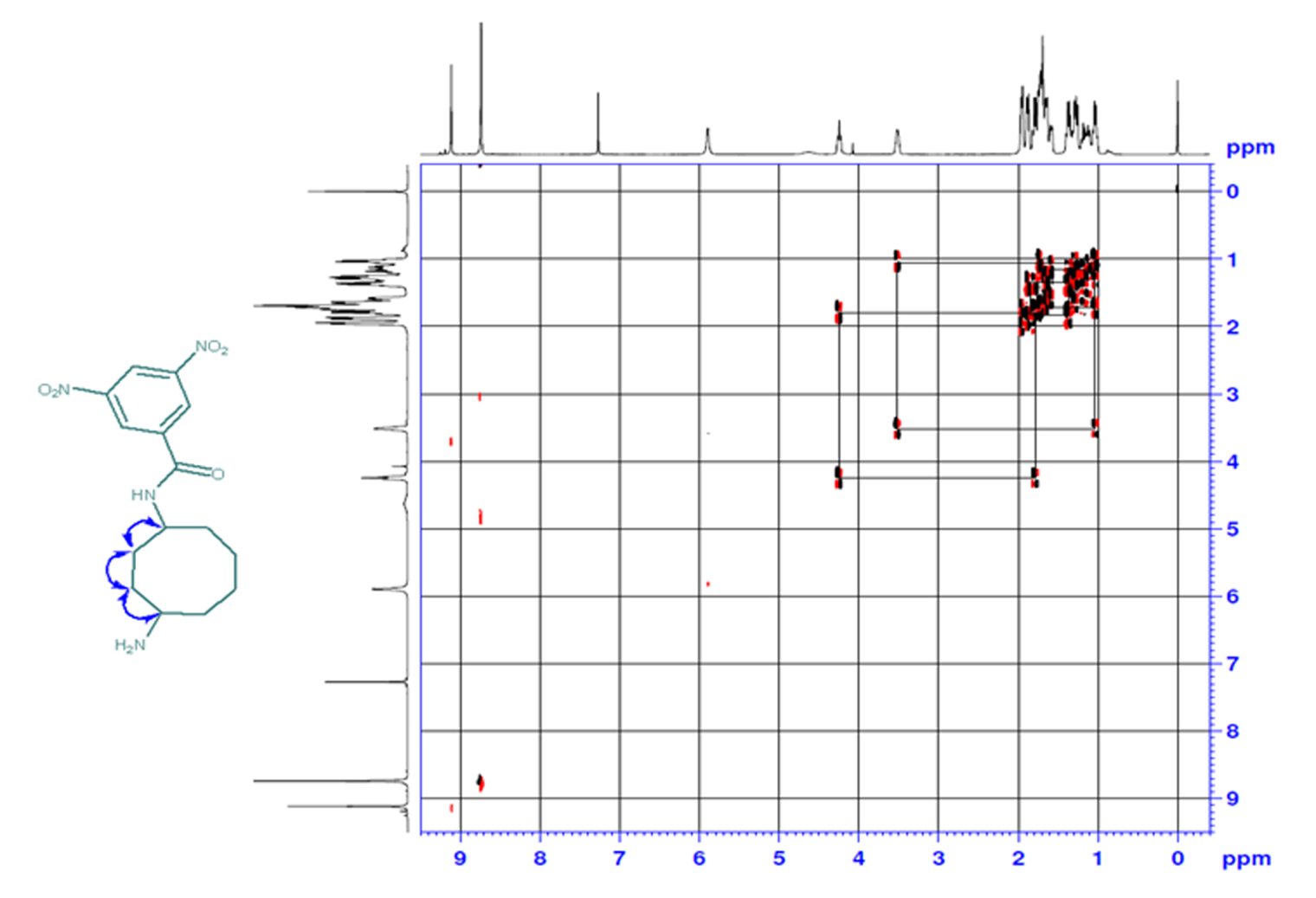


**Supplementary Fig. F:** ^1^H-^1^H COSY spectrum (CDCl_3_, 600 MHz) of N-(4-aminocyclooctyl) 3,5 dinitrobenzamide (1).


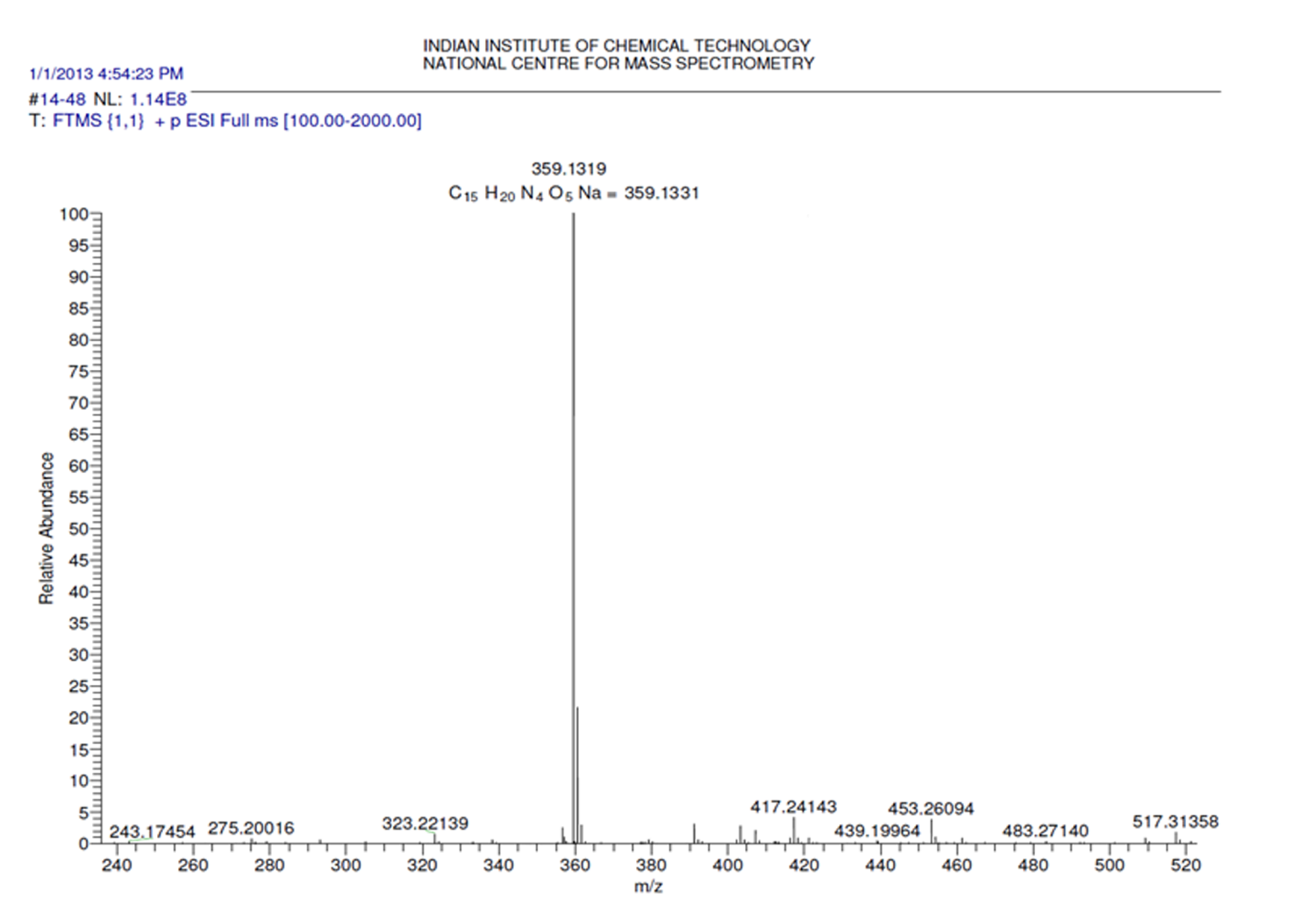


**Supplementary Fig. G:** HRESIMS spectrum of N-(4-aminocyclooctyl)-3, 5-dinitrobenzamide (**1**).


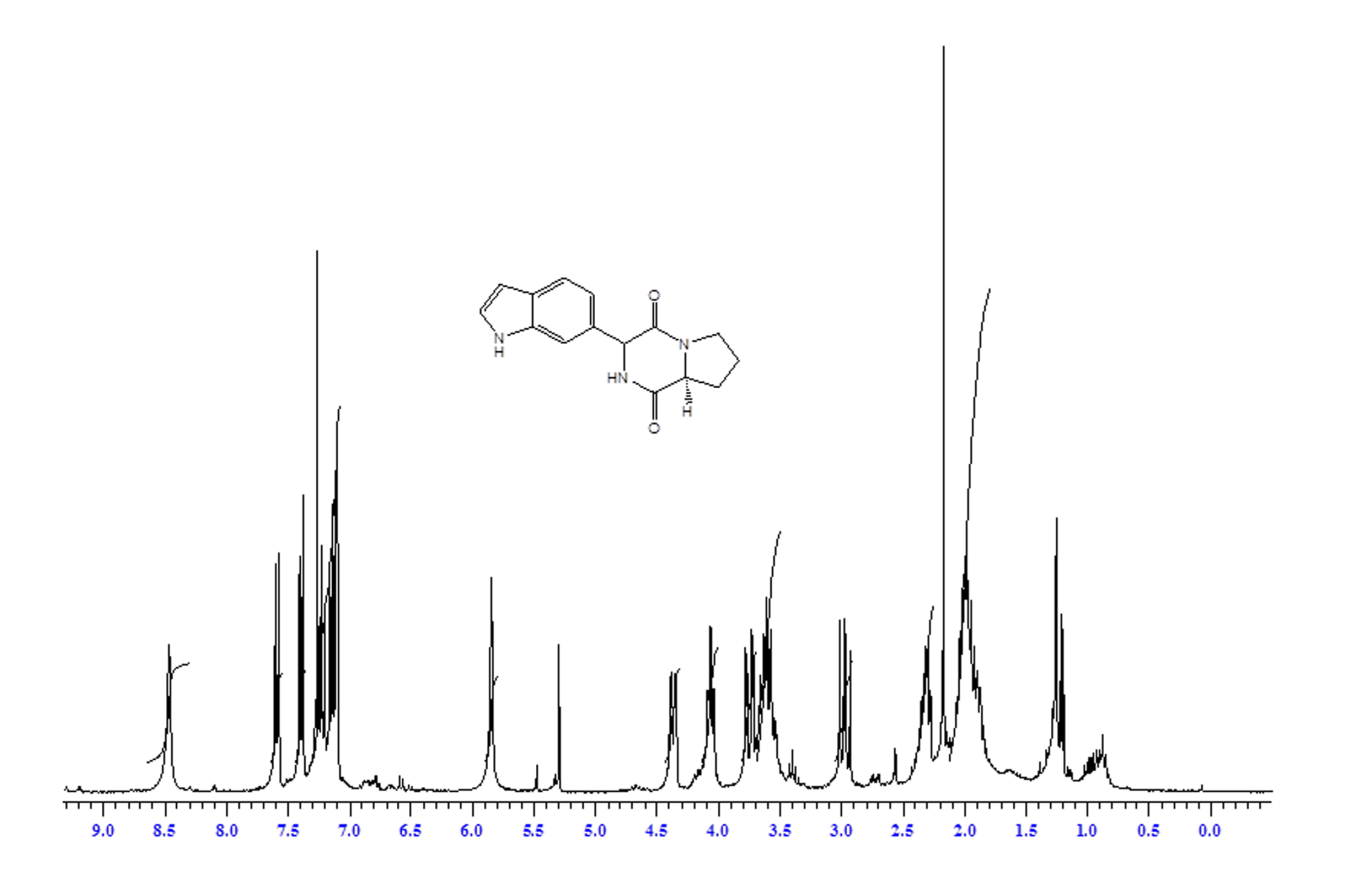


**Supplementary Fig. H:** ^1^H-NMR spectrum of the (3- ((1H-indol-6-yl) methyl) hexahydropyrrolo [1, 2-a] pyrazine-1, 4-dione (**2**).


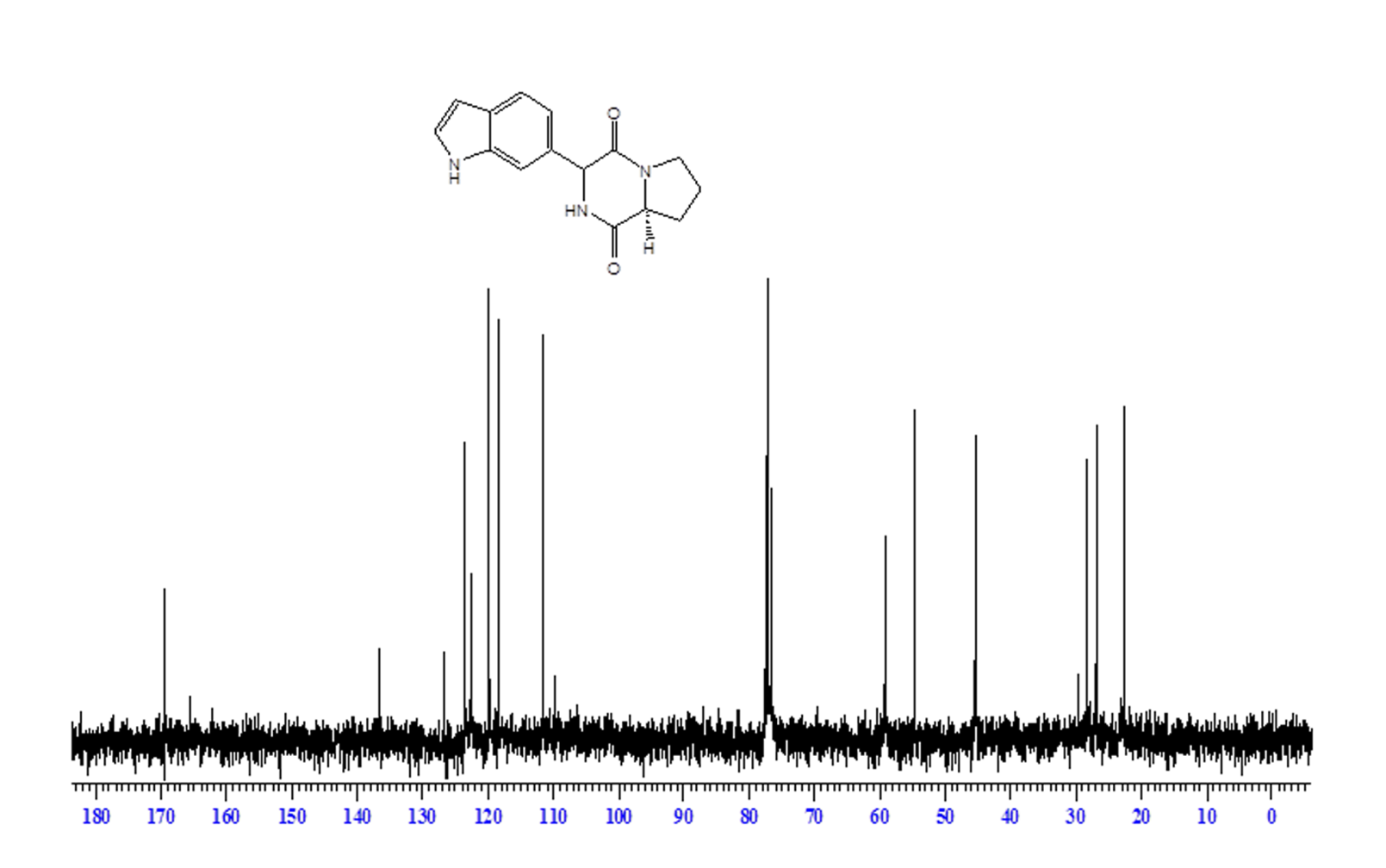


**Supplementary Fig. I:** ^13^C NMR spectrum of the (3- ((1H-indol-6-yl) methyl) hexahydropyrrolo [1, 2-a] pyrazine-1, 4-dione (**2**)


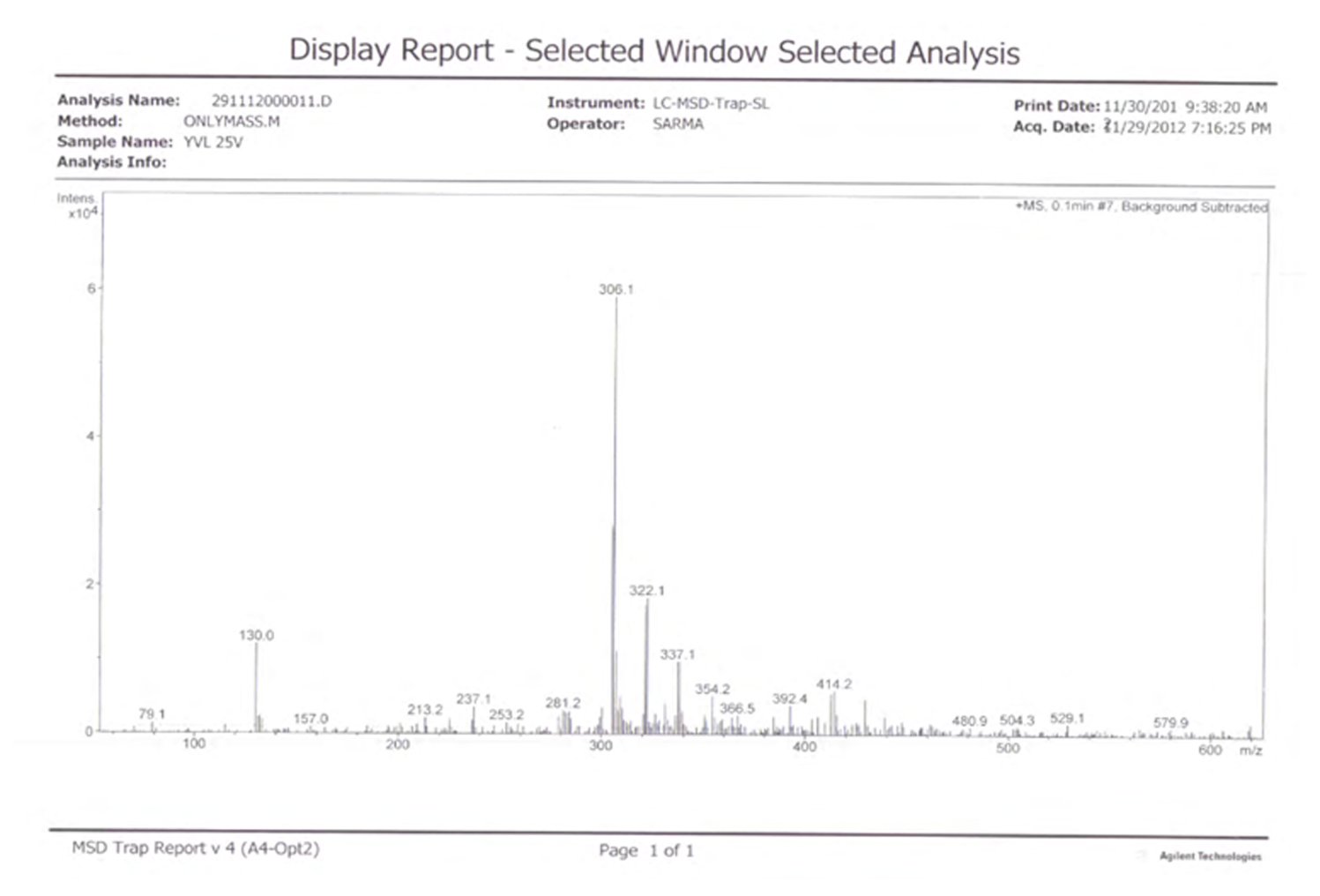


**Supplementary Fig. J:** Mass spectrum of the (3- ((1H-indol-6-yl) methyl) hexahydropyrrolo [1, 2-a] pyrazine-1, 4-dione (**2**)


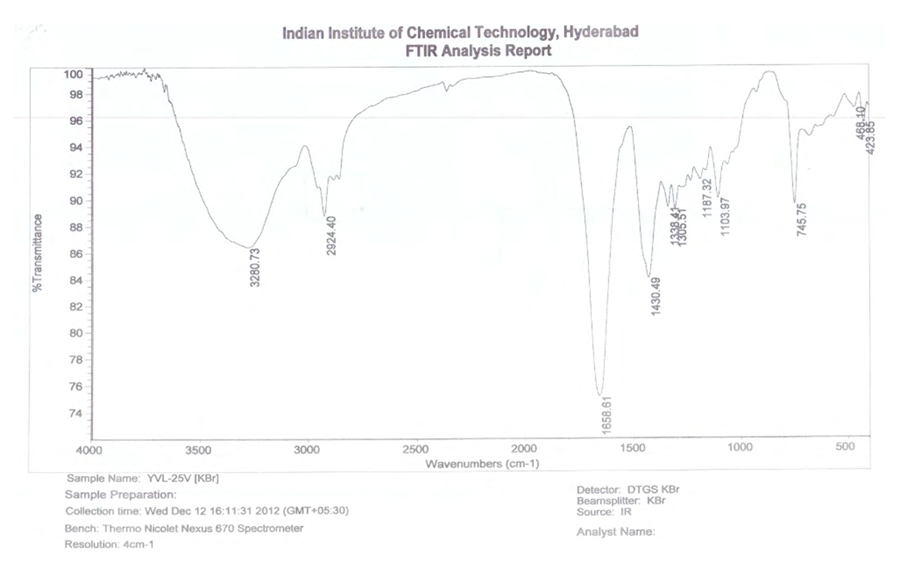


**Supplementary Fig. K:** FTIR spectrum of the (3- ((1H-indol-6-yl) methyl) hexahydropyrrolo [1, 2-a] pyrazine-1, 4-dione (**2**)
